# Knudsen Number Effects on Two-Dimensional Rayleigh–Taylor Instability in Compressible Fluid: Based on a Discrete Boltzmann Method

**DOI:** 10.3390/e22050500

**Published:** 2020-04-26

**Authors:** Haiyan Ye, Huilin Lai, Demei Li, Yanbiao Gan, Chuandong Lin, Lu Chen, Aiguo Xu

**Affiliations:** 1 College of Mathematics and Informatics, FJKLMAA, Fujian Normal University, Fuzhou 350117, China; haiyanye1227@163.com (H.Y.); dmli079@fjnu.edu.cn (D.L.); chenlu9801@163.com (L.C.); 2 North China Institute of Aerospace Engineering, Langfang 065000, China; phygan@163.com; 3 Sino-French Institute of Nuclear Engineering and Technology, Sun Yat-Sen University, Zhuhai 519082, China; 4 Laboratory of Computational Physics, Institute of Applied Physics and Computational Mathematics, P.O. Box 8009-26, Beijing 100088, China; Xu_Aiguo@iapcm.ac.cn; 5 State Key Laboratory of Explosion Science and Technology, Beijing Institute of Technology, Beijing 100081, China; 6 Center for Applied Physics and Technology, MOE Key Center for High Energy Density Physics Simulations, College of Engineering, Peking University, Beijing 100871, China

**Keywords:** discrete Boltzmann method, Rayleigh–Taylor instability, compressible fluid, Knudsen number, non-equilibrium effects

## Abstract

Based on the framework of our previous work [H.L. Lai et al., Phys. Rev. E, **94**, 023106 (2016)], we continue to study the effects of Knudsen number on two-dimensional Rayleigh–Taylor (RT) instability in compressible fluid via the discrete Boltzmann method. It is found that the Knudsen number effects strongly inhibit the RT instability but always enormously strengthen both the global hydrodynamic non-equilibrium (HNE) and thermodynamic non-equilibrium (TNE) effects. Moreover, when Knudsen number increases, the Kelvin–Helmholtz instability induced by the development of the RT instability is difficult to sufficiently develop in the later stage. Different from the traditional computational fluid dynamics, the discrete Boltzmann method further presents a wealth of non-equilibrium information. Specifically, the two-dimensional TNE quantities demonstrate that, far from the disturbance interface, the value of TNE strength is basically zero; the TNE effects are mainly concentrated on both sides of the interface, which is closely related to the gradient of macroscopic quantities. The global TNE first decreases then increases with evolution. The relevant physical mechanisms are analyzed and discussed.

## 1. Introduction

Rayleigh–Taylor (RT) instability is widespread in nature and industry. It arises that, when a light fluid supports or pushes a heavy one (that is, when there are acceleration points from a heavy density fluid to a light one), a physical phenomenon in which disturbances at the interface increase with time [1]. RT instability plays a very important role in many fields: for example, in the inertial confinement fusion (ICF) [2,3,4], the supernova explosion [5], the Z-pinch plasma [6], the quantum magnetized plasma [7], the colloid admixture [8], and so on. There has been a handful of reported experiments [9,10,11,12,13] to describe RT instability, and great results have been achieved. However, due to the harsh experimental conditions, many researchers prefer to resort to numerical approaches to study RT instability, such as the unified decomposition method [14], the flux-corrected transport [15], the finite element method [16], the front tracking method [17], the monte carlo method [18], the polyphase moving particle semi-implicit method [19], smoothed particle hydrodynamics method [20], etc. In 1988, Gr$\overset{´}{e}$tar et al. proposed a two-way coupled Euler–Lagrange method to simulate the motion of non-viscous fluid interfaces with different densities, and it was applied to the study of RT instability [21]. In 2004, Miles et al. studied the effect of initial conditions on the two-dimensional (2D) RT instability and excessive turbulence of a planar detonation wave drive system, and obtained that the existence of short-wavelength components plays an important role in turbulent transitions, but its effect on large scale does not depend on its spectral properties [22]. In 2009, Banerjee et al. used the direct numerical simulation to study the RT instability of three-dimensional (3D) incompressible miscible fluids [23]. In 2012, Ye et al. considered non-spin incompressible inviscid fluids and analyzed the RT instability of the discontinuous distribution of the interface by analytical methods [24]. In 2015, Rozanov et al. studied the effect of initial conditions on RT instability, and it was obtained that the initial disturbance promoted the formation of a large number of 2D structures in the fluid [25]. In the same year, Sagert et al. used a modified direct simulation Monte Carlo method to study single-mode RT instability for a large range of Knudsen numbers [26]. In 2016, Yan et al. studied the nonlinear evolution of single-mode ablation RT instability in the 3D case, when the mode wavelength approaches the cutoff point of the linear spectrum, they found that the velocity of 3D saturated bubble is much faster than that of 2D bubble and classical 3D bubble [27]. In the same year, Zhou et al. used a numerical method to analyze the statistics of the kinetic energy and thermal energy dissipation rate of 2D RT turbulence [28]. Aslangil et al. used an implicit large eddy simulation technique to analyze the effect of initial conditions on the stability of miscible incompressible barotropic pressure driven RT under non-uniform acceleration conditions [29]. In 2018, Zhang et al. studied the instability of 2D and 3D multimode ablation RT. The simulation results showed that, under the condition of the same initial disturbance amplitude, the instability of nonlinear ablation RT was controlled by bubble competition. Compared with the classical scenario, a large amount of ablation could reduce the growth of nonlinear bubbles [30]. In 2019, based on compressible Navier–Stokes (NS) equations, Hu et al. studied the effect of viscosity on 2D single-mode RT instability during and after the re-acceleration stage [31]. In the same year, Kord et al. adopted a discrete-based adjoint method to control multimode RT instability by controlling the disturbance at the initial interface [32]. Liu et al. considered the single-mode classical RT instability of any Atwood number in the weakly nonlinear stage and studied the effect of mode coupling branches on harmonic amplitudes by means of modal coupling tracking technology [33]. Doludenko et al. analyzed the RT instability in the inviscous and viscous situations by performing direct numerical simulations of the Euler equation and the NS equations [34]. In 2020, Luo et al. used the finite difference method to simulate the late-time evolution of single-mode RT instability for isothermal stratification to study effects of compressibility and Atwood number [35]. These studies provide a lot of useful information for understanding the physical mechanism of RT instability [36,37].

In terms of mesoscopic numerical simulation, the lattice Boltzmann (LB) method, originally developed from a special discrete form of Boltzmann equation, has been widely used to solve corresponding hydrodynamic equations in various complex fluids, such as incompressible flows [38,39,40,41], porous media [42,43,44], magnetohydrodynamics [45,46], nonlinear wave [47,48], microchannel flow [49,50,51], nanofluid [52], and multiphase flows [53]. There were also many LB works to study the RT instability, and some useful results were obtained. In 1999, He et al. proposed an LB scheme and simulated the RT instability of the multiphase flow in the nearly incompressible limit [54]. In 2010, Scagliarini et al. used a thermal LB model to study RT instability of compressible stratified flows [55]. In 2012, Li et al. studied incompressible multiphase flows by LB model with additional interfacial force, and numerical simulations were carried out including the RT instability [56]. In 2015, Wang et al. proposed a multiphase LB flux solver for incompressible multiphase flows with low and large density ratios, which is validated by several benchmark problems, including RT instability [57]. In 2016, Liang et al. investiged the LB simulation of 3D RT instability and studied the effect of Reynolds number on the interfacial dynamic and spike and bubble amplitudes in detail [58]. In 2017, Wei et al. proposed an efficient coupled LB model for incompressible flow in application of turbulence RT instability [59]. The next year, Yang et al. studied the behavior of thermal and viscous entropy generation of global quantities with time evolution in RT turbulence mixing through the LB method [60]. In 2019, Liang et al. used an incompressible LB model to study multi-mode immiscible RT instability with high Reynolds numbers [61].

To our knowledge, the above studies mainly focus on the hydrodynamic non-equilibrium (HNE) effects of RT instability, and neglect the thermodynamic non-equilibrium (TNE) effects during the evolution of RT instability. To better describe the TNE effects on RT instability, we turn to the discrete Boltzmann method (DBM) [62,63,64] which is from coarse-grained modeling of the Boltzmann equation. The coarse-grained modeling is a process where the most important elements are kept and others may be neglected according to physical problem under study. The physical quantities that we will use to describe the system behavior must keep the same values after the simplification. From molecular dynamics or Liouville equation to Boltzmann’s equation, to the Burnett, the NS equations and Euler equations, coarse-grained modeling is a process of gradually neglecting detailed information which become relatively less important with increasing the spatio-temporal scale used to investigate the system. Over the past years, the DBM has been efficiently applied to many fields and brought some new insights into the corresponding systems. For example, in 2017, Lin et al. proposed a multi-component DBM for premixed, nonpremixed, or partially premixed non-equilibrium reactive flows, and the model is suitable for both subsonic and supersonic flows with or without chemical reaction and/or external force [65]. In 2018, Xu et al. presented a theoretical framework for constructing a DBM with spherical symmetry in spherical coordinates to kinetically model implosion- and explosion-related phenomena [66]. Lin et al. employed the DBM to study the HNE and TNE effects of the chemical reactant around the detonation wave [67]. Zhang et al. proposed the discrete ellipsoidal statistical Bhatnagar–Gross–Krook (BGK) model to simulate non-equilibrium compressible flows with a flexible Prandtl number [68]. In 2019, Gan et al. studied the effects of viscosity and heat conduction on the Kelvin–Helmholtz (KH) instability through the DBM. The authors found the viscosity effects stabilize the KH instability and enhance both the local and global TNE intensities [69]. Lin et al. employed the DBM to investigate the KH instability and found the relaxation time always strengthens the global non-equilibrium, entropy of mixing, and free enthalpy of mixing [70]. Besides by theory, results of DBM have been confirmed and supplemented by results of molecular dynamics [71,72], direct simulation Monte Carlo [73], and experiment [65].

In recent studies, compressible RT instability is studied by the DBM. In 2016, Lai et al. investigated the interaction between HNE and TNE effects using the DBM, and studied the effect of compressibility on 2D RT instability [74]. In the same year, Chen et al. used a multi-relaxation-time DBM to investigate the effects of viscosity, heat conductivity, and Prandtl number on the 2D RT instability from macroscopic and non-equilibrium viewpoints, and found that viscosity and heat conduction suppress RT instability mainly by suppressing the re-acceleration phase KH instability [75]. In 2017, Lin et al. extended the DBM to the compressible system containing two components with independent specific-heat ratios, and studied the dynamic process of the RT instability with two components [76]. In 2018, Chen et al. adopted a multi-relaxation-time DBM to numerically simulate a 2D Richtmyer–Meshkov (RM) instability and RT instability coexisting system [77]. Around the same time, Li et al. used the DBM to study the multi-mode RT instability with discontinuous interface in a compressible flow [78]. In 2019, Zhang et al. made a Lagrangian tracking supplement to the DBM, and studied the RT instability of the two-miscible-fluid system [79]. Despite the aforementioned efforts, there are still many problems needed to be studied. Based on previous work [74], in the following, we focus on the HNE and TNE behaviors, and aim to demonstrate the effects of the Knudsen number on the onset and growth of the 2D RT instability.

The content of this paper is organized as follows. In the next section, we briefly illustrate the DBM used in this work. The HNE and TNE behaviors about the 2D RT instability and the effects of Knudsen number are studied in Section 3. Finally, the research conclusions of this paper have been summarized and its further studies forecasted.

## 2. Discrete Boltzmann Model

The Boltzman equation of BGK collision with an external force [80] is:

(1) $$\partial_{t}f+v\cdot \nabla_{r}f+a\cdot \nabla_{v}f=-\frac{1}{\tau }\left(f-f^{eq}\right),$$

where f(r,v,t) represents the distribution function of a single particle, r the spatial coordinates, v the velocity of particle, *t* the time, τ the relaxation time, a the external force, and $f^{eq}$ the local equilibrium distribution function. The expression of $f^{eq}$ is as follows [81]:

(2) $$f^{eq}=\rho {\left(\frac{1}{2\pi T}\right)}^{D/2}{\left(\frac{1}{2\pi nT}\right)}^{1/2}\exp\left[-\frac{{\left(v-u\right)}^{2}}{2T}-\frac{\eta^{2}}{2nT}\right],$$

where η is a free parameter, *D* is the dimension of space, ρ, *T*, and u represent density, temperature, and fluid velocity, respectively. *n* is the number of extra degrees of freedom corresponding to molecular rotation and/or internal vibration.

In the Boltzmann equation with the BGK collision term, the particle velocity distribution function is replaced by the discrete velocity distribution function. It can be assumed that the system is very close to the equilibrium state, so *f* in the external term can be replaced by $f^{eq}$. Then, we can obtain the discrete Boltzmann equation with an external force term:

(3) $$\frac{\partial f_{i}}{\partial t}+v_{i}\cdot \frac{\partial f_{i}}{\partial r}-\frac{a\cdot (v_{i}-u)}{T}f_{i}^{eq}=-\frac{1}{\tau }\left(f_{i}-f_{i}^{eq}\right),$$

where $f_{i}$ and $f_{i}^{eq}$ represent the discrete distribution function and discrete equilibrium distribution function corresponding to the *i*-th discrete velocity $v_{i}$, with i=1,2,⋯,N. The discrete local equilibrium distribution function $f_{i}^{eq}$ should satisfy the following seven relationships:

(4) $$\begin{array}{c} \;\int \int \;f^{eq}dvd\eta =\sum_{i}f_{i}^{eq}=\rho , \end{array}$$

(5) $$\begin{array}{c} \;\int \int \;f^{eq}vdvd\eta =\sum_{i}f_{i}^{eq}v_{i}=\rho u, \end{array}$$

(6) $$\begin{array}{c} \;\int \int \;f^{eq}\left(v\cdot v+\eta^{2}\right)dvd\eta =\sum_{i}f_{i}^{eq}\left(v_{i}\cdot v_{i}+\eta_{i}^{2}\right)=\rho \left[(D+n)T+u\cdot u\right], \end{array}$$

(7) $$\begin{array}{c} \;\int \int \;f^{eq}vvdvd\eta =\sum_{i}f_{i}^{eq}v_{i}v_{i}=\rho \left(TI+uu\right), \end{array}$$

(8) $$\begin{array}{c} \;\int \int \;f^{eq}\left(v\cdot v+\eta^{2}\right)vdvd\eta =\sum_{i}f_{i}^{eq}\left(v_{i}\cdot v_{i}+\eta_{i}^{2}\right)v_{i}=\rho u\left[(D+n+2)T+u\cdot u\right], \end{array}$$

(9) $$\begin{array}{c} \;\int \int \;f^{eq}vvvdvd\eta =\sum_{i}f_{i}^{eq}v_{i}v_{i}v_{i}=\rho \left[T(u_{\alpha }e_{\beta }e_{\chi }\delta_{\beta \chi }+e_{\alpha }u_{\beta }e_{\chi }\delta_{\alpha \chi }+e_{\alpha }e_{\beta }\delta_{\alpha \beta }u_{\chi })+uuu\right], \end{array}$$

(10) $$\begin{array}{c} \;\int \int \;f^{eq}\left(v\cdot v+\eta^{2}\right)vvdvd\eta =\sum_{i}f_{i}^{eq}\left(v_{i}\cdot v_{i}+\eta_{i}^{2}\right)v_{i}v_{i}=\rho T\left[(D+n+2)T+u\cdot u\right]e_{\alpha }e_{\beta }\delta_{\alpha \beta }\\ +\rho uu\left[(D+n+4)T+u\cdot u\right], \end{array}$$

where $\delta_{\alpha \beta }$ is Kronecker delta function, and α, β indicate either the *x* or *y* component. $e_{\alpha }$ is the unit vector in the α direction.

The above seven relationships can be written in a matrix form as follows (the boldface symbols denote *N*-dimensional column vectors):

(11) $$\begin{array}{c} C\cdot f^{eq}={\hat{f}}^{eq}, \end{array}$$

where

(12) $$\begin{array}{c} C=\left(C_{1},C_{2},\cdots ,C_{N}\right),C_{i}={\left(c_{i1},c_{i2},\cdots ,c_{iN}\right)}^{T}, \end{array}$$

(13) $$\begin{array}{c} f^{eq}={\left[f_{1}^{eq},f_{2}^{eq},\cdots ,f_{N}^{eq}\right]}^{T}, \end{array}$$

(14) $$\begin{array}{c} {\hat{f}}^{eq}={\left[{\hat{f}}_{1}^{eq},{\hat{f}}_{2}^{eq},\cdots ,{\hat{f}}_{N}^{eq}\right]}^{T}, \end{array}$$

where N=16 in two dimensions in the present model, $c_{i1}=1$, $c_{i2}=v_{ix}$, $c_{i3}=v_{iy}$, $c_{i4}=v_{ix}^{2}+v_{iy}^{2}+\eta_{i}^{2}$, $c_{i5}=v_{ix}v_{iy}$, $c_{i6}=v_{ix}^{2}$, $c_{i7}=v_{iy}^{2}$, $c_{i8}=v_{ix}\left(v_{ix}^{2}+v_{iy}^{2}+\eta_{i}^{2}\right)$, $c_{i9}=v_{iy}\left(v_{ix}^{2}+v_{iy}^{2}+\eta_{i}^{2}\right)$, $c_{i10}=v_{ix}^{3}$, $c_{i11}=v_{iy}^{3}$, $c_{i12}=v_{ix}^{2}v_{iy}$, $c_{i13}=v_{ix}v_{iy}^{2}$, $c_{i14}=v_{ix}v_{iy}\left(v_{ix}^{2}+v_{iy}^{2}+\eta_{i}^{2}\right)$, $c_{i15}=v_{ix}^{2}\left(v_{ix}^{2}+v_{iy}^{2}+\eta_{i}^{2}\right)$, $c_{i16}=v_{iy}^{2}\left(v_{ix}^{2}+v_{iy}^{2}+\eta_{i}^{2}\right)$. ${\hat{f}}_{1}^{eq}=\rho$, ${\hat{f}}_{2}^{eq}=\rho u_{x}$, ${\hat{f}}_{3}^{eq}=\rho u_{y}$, ${\hat{f}}_{4}^{eq}=\rho \left[\left(D+n\right)T+u_{x}^{2}+u_{y}^{2}\right]$, ${\hat{f}}_{5}^{eq}=\rho u_{x}u_{y}$, ${\hat{f}}_{6}^{eq}=\rho \left(T+u_{x}^{2}\right)$, ${\hat{f}}_{7}^{eq}=\rho \left(T+u_{y}^{2}\right)$, ${\hat{f}}_{8}^{eq}=\rho u_{x}\left[\left(D+n+2\right)T+u_{x}^{2}+u_{y}^{2}\right]$, ${\hat{f}}_{9}^{eq}=\rho u_{y}\left[\left(D+n+2\right)T+u_{x}^{2}+u_{y}^{2}\right]$, ${\hat{f}}_{10}^{eq}=\rho u_{x}\left(3T+u_{x}^{2}\right)$, ${\hat{f}}_{11}^{eq}=\rho u_{y}\left(3T+u_{y}^{2}\right)$, ${\hat{f}}_{12}^{eq}=\rho u_{y}\left(T+u_{x}^{2}\right)$, ${\hat{f}}_{13}^{eq}=\rho u_{x}\left(T+u_{y}^{2}\right)$, ${\hat{f}}_{14}^{eq}=\rho u_{x}u_{y}\left[\left(D+n+4\right)T+u_{x}^{2}+u_{y}^{2}\right]$, ${\hat{f}}_{15}^{eq}=\rho T\left[\left(D+n+2\right)T+u_{x}^{2}+u_{y}^{2}\right]+\rho u_{x}^{2}\left[\left(D+n+4\right)T+u_{x}^{2}+u_{y}^{2}\right]$, ${\hat{f}}_{16}^{eq}=\rho T\left[\left(D+n+2\right)T+u_{x}^{2}+u_{y}^{2}\right]+\rho u_{y}^{2}\left[\left(D+n+4\right)T+u_{x}^{2}+u_{y}^{2}\right]$.

If the inverse of the matrix C exists, then the discrete equilibrium distribution function is expressed as:

(15) $$\begin{array}{c} f^{eq}=C^{-1}\cdot {\hat{f}}^{eq}, \end{array}$$

so the choice of discrete velocity model (DVM) has a high degree of flexibility which results in higher efficiency and better stability of this approach [63].

According to the conservations of mass, momentum, and energy, we have the following relationships:

(16) $$\begin{array}{c} \sum_{i}f_{i}=\sum_{i}f_{i}^{eq}=\rho , \end{array}$$

(17) $$\begin{array}{c} \sum_{i}f_{i}v_{i}=\sum_{i}f_{i}^{eq}v_{i}=\rho u, \end{array}$$

(18) $$\begin{array}{c} \sum_{i}f_{i}\left(v_{i}\cdot v_{i}+\eta_{i}^{2}\right)=\sum_{i}f_{i}^{eq}\left(v_{i}\cdot v_{i}+\eta_{i}^{2}\right)=\rho \left[(D+n)T+u\cdot u\right]. \end{array}$$

By means of the Chapman–Enskog multiscale analysis, we can get the compressible NS equations from the DBM as follows:

(19) $$\begin{array}{c} \left\{\begin{array}{c} \frac{\partial \rho }{\partial t}+\frac{\partial (\rho u_{\alpha })}{\partial r_{\alpha }}=0,\\ \frac{\partial }{\partial t}\left(\rho u_{\alpha }\right)+\frac{\partial }{\partial r_{\beta }}\left(p\delta_{\alpha \beta }+\rho u_{\alpha }u_{\beta }\right)-\frac{\partial p_{\alpha \beta }^{{}^{\prime }}}{\partial r_{\beta }}=\rho a_{\alpha },\\ \frac{\partial }{\partial t}\left[\rho \left(e+\frac{1}{2}u^{2}\right)\right]+\frac{\partial }{\partial r_{\alpha }}\left[\rho u_{\alpha }\left(e+\frac{1}{2}u^{2}\right)+Pu_{\alpha }\right]=\frac{\partial }{\partial r_{\alpha }}\left(\kappa \frac{\partial T}{\partial r_{\alpha }}+u_{\beta }p_{\alpha \beta }^{\prime }\right)+\rho u_{\alpha }a_{\alpha }, \end{array}\right. \end{array}$$

where

(20) $$\begin{array}{c} p_{\alpha \beta }^{{}^{\prime }}=\mu \left(\frac{\partial u_{\alpha }}{\partial r_{\beta }}+\frac{\partial u_{\beta }}{\partial r_{\alpha }}-\frac{2}{D+n}\frac{\partial u_{\chi }}{\partial r_{\chi }}\delta_{\alpha \beta }\right), \end{array}$$

and the pressure is

(21) $$\begin{array}{c} p=\rho T, \end{array}$$

the total internal energy is

(22) $$\begin{array}{c} e=\frac{D+n}{2}T, \end{array}$$

the dynamic viscosity coefficient is

(23) $$\begin{array}{c} \mu =p\tau , \end{array}$$

the thermal conductivity viscosity coefficient is

(24) $$\begin{array}{c} \kappa =\frac{D+n+2}{2}p\tau . \end{array}$$

In this way, we obtain the distribution function $f_{i}$ by solving the discrete Boltzmann Equation (3), then get the macro quantity ρ through Equation (16), get the macro quantity u by Equation (17), and get the macro quantity *T* by Equation (18).

**Figure 1:**
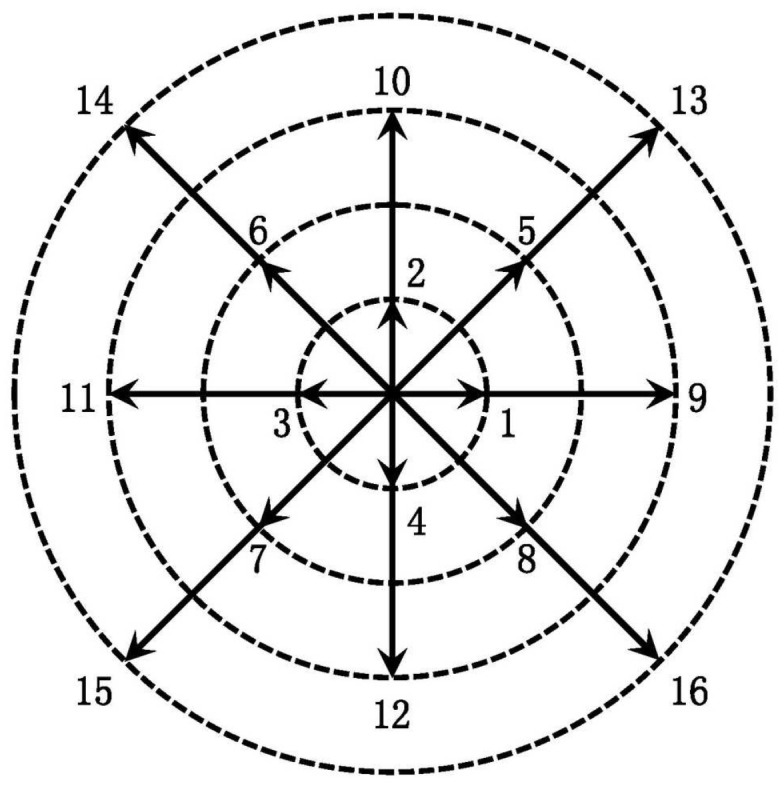
Schematic diagram of D2V16 for DVM.

Here, the following DVM (D2V16) is adopted:

(25) $$v_{i}=\left\{\begin{array}{cc} c\left[\cos\frac{(i-1)\pi }{2},\sin\frac{(i-1)\pi }{2}\right], & i=1,\cdots ,4,\\ 2c\left[\cos\frac{(2i-1)\pi }{4},\sin\frac{(2i-1)\pi }{4}\right], & i=5,\cdots ,8,\\ 3c\left[\cos\frac{(i-9)\pi }{2},\sin\frac{(i-9)\pi }{2}\right], & i=9,\cdots ,12,\\ 4c\left[\cos\frac{(2i-9)\pi }{4},\sin\frac{(2i-9)\pi }{4}\right], & i=13,\cdots ,16, \end{array}\right.$$

where i=1,2,⋯,4, $\eta_{i}=\eta_{0}$, and i=5,6,⋯,16, $\eta_{0}=0$. *c* is the magnitude of the particle velocity in the first circle. The DVM of D2V16 is shown as Figure 1.

The DVM of D2V16 gets rid of the binding between spatial and time discretization, makes the particle velocity flexible, and it is convenient to introduce various computational methods to solve the discrete Boltzmann equation (3). The DBM is considered to be a special discrete form of the Boltzmann equation, and naturally inherits the properties of Boltzmann equation that can be used to describe TNE effects. Among the seven kinetic moment relations (4)–(10), only for the first three ones, $f_{i}^{eq}$ can be replaced by $f_{i}$. This is because, in or out of the equilibrium, the mass, momentum, and energy conservations are kept. However, the other four kinetic moment relations, if $f_{i}^{eq}$ is replaced by $f_{i}$, the two sides of these equations might not be equal, and some deviations will appear. From a physical point of view, these deviations are caused by the system deviating from the local equilibrium state. In this paper, we consider the TNE effects of the thermal fluctuation characteristics of micro-particles with macroscopic flow removed. The corresponding center moment $M_{m,n}^{*}$ are defined as:

(26) $$\begin{array}{c} \left\{\begin{array}{c} M_{2}^{*}\left(f_{i}\right)=\sum_{i}f_{i}v_{i}^{*}v_{i}^{*},\\ M_{3}^{*}\left(f_{i}\right)=\sum_{i}f_{i}v_{i}^{*}v_{i}^{*}v_{i}^{*},\\ M_{3,1}^{*}\left(f_{i}\right)=\sum_{i}f_{i}\left(v_{i}^{*}\cdot v_{i}^{*}+\eta_{i}^{2}\right)v_{i}^{*},\\ M_{4,2}^{*}\left(f_{i}\right)=\sum_{i}f_{i}\left(v_{i}^{*}\cdot v_{i}^{*}+\eta_{i}^{2}\right)v_{i}^{*}v_{i}^{*}, \end{array}\right. \end{array}$$

where $v_{i}^{*}=v_{i}-u$. In order to qualitatively analyze the evolution of TNE effects, we further define the total average TNE effects or intensity:

(27) $$\begin{array}{c} D^{*}=\sqrt{|{\bar{\Delta }}_{2}^{*}{|}^{2}+|{\bar{\Delta }}_{3}^{*}{|}^{2}+|{\bar{\Delta }}_{3,1}^{*}{|}^{2}+{\left|{\bar{\Delta }}_{4,2}^{*}\right|}^{2}}, \end{array}$$

where ${\bar{\Delta }}_{m,n}^{*}$ represents the absolute average of the full field for each TNE effect which is defined as follows:

(28) $$\begin{array}{ccc} \Delta_{m,n}^{*} & = & M_{m,n}^{*}\left(f_{i}\right)-M_{m,n}^{*}\left(f_{i}^{eq}\right). \end{array}$$

## 3. Numerical Simulations

In this section, we focus on the 2D compressible RT instability. The starting configuration is in a 2D domain [−d/2,d/2]×[−2d,2d]. The entire system is in a gravitational field with constant gravitational acceleration, and the initial disturbance of the interface satisfies $y_{c}\left(x\right)=y_{0}\cos\left(kx\right)$, *k* is the wave number and k=2π/λ, λ is the wavelength. We create different layers of density by setting different temperatures. The temperatures of two half layers are initially set to constant, then the system satisfies by the hydrostatic equilibrium condition:

(29) $$\partial_{y}p_{0}\left(y\right)=-g\rho_{0}\left(y\right),$$

and therefore we have the initial hydrostatic unstable condition as:

(30) $$\left\{\begin{array}{c} T_{0}\left(y\right)=T_{u},\;\rho_{0}\left(y\right)=\frac{p_{0}}{T_{u}}\exp\left[\frac{g}{T_{u}}\left(2d-y\right)\right],y>y_{c}\left(x\right),\\ T_{0}\left(y\right)=T_{b},\;\rho_{0}\left(y\right)=\frac{p_{0}}{T_{b}}\exp\left[\frac{g}{T_{u}}\left(2d-y_{c}\left(x\right)\right)-\frac{g}{T_{b}}\left(y-y_{c}\left(x\right)\right)\right],y<y_{c}\left(x\right), \end{array}\right.$$

where $p_{0}$ is the pressure at the top boundary at the beginning. $T_{u}$ and $T_{b}$ are the constant temperatures of the upper and bottom half parts, respectively. On this condition, we have the same static pressure at the disturbance interface:

(31) $$\rho_{u}T_{u}=\rho_{b}T_{b},$$

where $\rho_{u}$ and $\rho_{b}$ are the densities of the upper and bottom grid aside the disturbance interface. Furthermore, the initial Atwood number is defined as follows:

(32) $$At=\frac{\rho_{u}-\rho_{b}}{\rho_{u}+\rho_{b}}=\frac{T_{b}-T_{u}}{T_{b}+T_{u}}.$$

In our simulations, the boundary conditions used in this work are as follows: the adiabatic and non-slip boundary conditions are used for the upper and lower boundaries; and symmetrical boundary conditions are used for the left and right sides.

Here, as in the previous work [74], we choose the units of length and time as 1/k and ${\left(gk\right)}^{-1/2}$, respectively, then the unit of velocity is ${\left(g/k\right)}^{1/2}$, so we have two dimensionless parameters as follows:

(33) $$H_{1}=\frac{g/k}{c_{s}^{2}},$$

and

(34) $$H_{2}=\tau \sqrt{gk}=\frac{\tau }{{\left(gk\right)}^{-1/2}},$$

where $H_{1}$ describes the compressibility of the flow, $H_{2}$ describes the ratio of the relaxation time, and a macroscopic time. It can be deduced obviously that $H_{2}$ can represent the Knudsen number. In addition, $H_{2}$ works as a τ parameter to roughly describe how far the system deviates from its thermodynamic equilibrium in the initial state. In addition, not only that, $H_{2}$ also describes the relative strength of dissipation work rate over mechanical work rate. To study the Knudsen number $H_{2}$, we should make sure the compressibility number $H_{1}$ is constant.

In the present work, the calculation area we adopted is a uniform grid of $N_{x}\times N_{y}=256\times 1024$, the space step $\Delta x=\Delta y=10^{-3}$, the time step $\Delta t=10^{-5}$, then $k=2\pi /\lambda =2\pi /(N_{x}\times \Delta x)=49.0874$. The initial pressure at the top $p_{0}=1.0$, the upper part temperature and the lower part temperature are $T_{u}=1.0$, $T_{b}=4.0$, then $A_{t}=0.6$. Other parameters are c=1.3, η=15, n=3, $a_{x}=0.0$, $a_{y}=-g=-1.0$. We calculate the value of $c_{s}$ by the initial temperature $T_{u}$ in the upper part as $c_{s}=\sqrt{\gamma T_{u}}=\sqrt{1.4\times 1}=1.1832$, then we can calculate the compressibility $H_{1}=1.4551\times 10^{-2}$. We can change the relaxation time to change the Kundsen number $H_{2}$.

**Figure 2:**
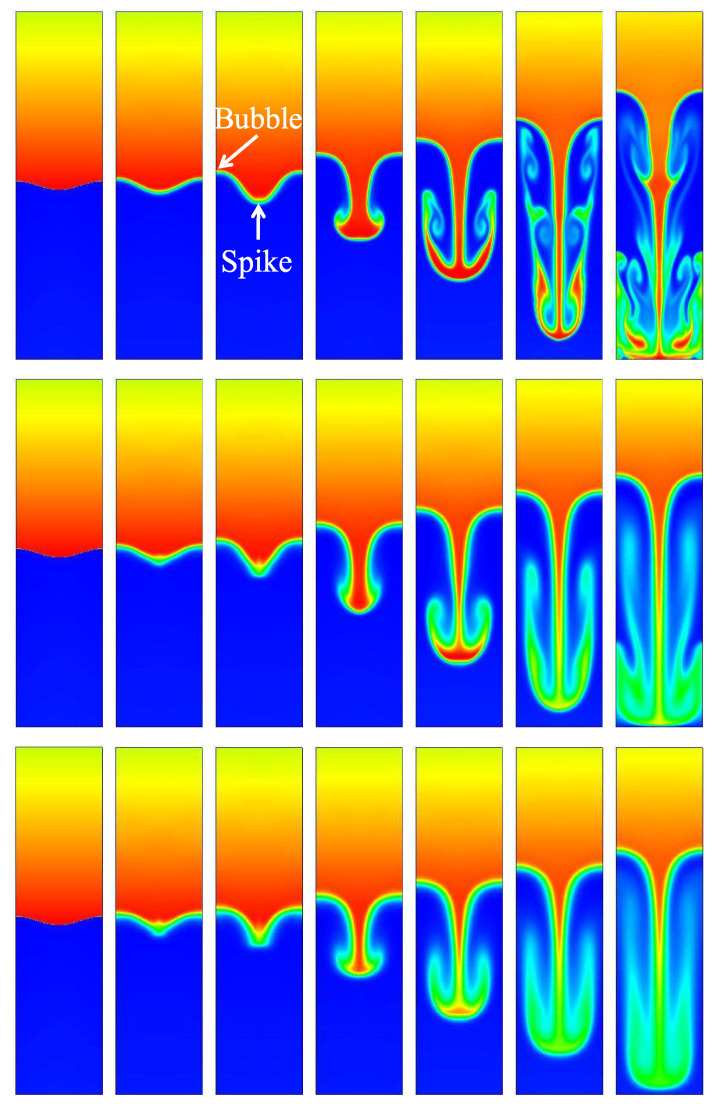
Evolution of the density contour at time instants (from left to right) t=0.0, 0.2, 0.4, 0.8, 1.2, 1.6, and 2.0, respectively. The three rows, from top to bottom, correspond to the cases with Knudsen numbers $H_{2}=7.0062\times 10^{-5}$, $H_{2}=2.1019\times 10^{-4}$, and $H_{2}=3.5031\times 10^{-4}$, respectively. When $H_{2}$ increases, the inhibition effect becomes more obvious.

In order to observe the evolution of the 2D RT instability with time more intuitively, the density evolution is shown in Figure 2. We choose three relaxation times: $\tau =1\times 10^{-5}$, $3\times 10^{-5}$, $5\times 10^{-5}$, corresponding to three Knudsen numbers: $H_{2}=7.0062\times 10^{-5}$, $H_{2}=2.1019\times 10^{-4}$ and $H_{2}=3.5031\times 10^{-4}$, respectively. These simulation results show that, for all $H_{2}$ values, due to the effects of the gravity, the disturbance interface is rolled up at the tip or peak, and then two pairs of opposite vortices are formed. The perturbation interfaces all evolved into the shapes of “bubble” and “spike”. Here, the structure of rising lighter fluid is referred to as bubble, and the structure of falling heavier fluid is referred to as spike. We also find that the smaller the $H_{2}$, the faster the roll-up phenomenon appears. Moreover, the smaller with $H_{2}$ value, the stronger influence of KH instability on the later stage, the vortex will further develop, become longer, and form two pairs of long rolls, finer structures appear. When the spike approaches the lower wall, the lower part of the bubble is deflected downward while the upper part keeps moving up, maintaining the “mushroom” shape. As the $H_{2}$ value increases, the size of the vortex decreases significantly, these rolls squeeze the light fluid inward, and then contact the heavy fluid, the spike tail is flipped up to form a mushroom shape, at which time the nonlinear effect is very strong. At the same time, we observe that, in the initial stage, the thermal diffusion quickly smoothes the discontinuous, and the larger $H_{2}$, the stronger thermal diffusion.

**Figure 3:**
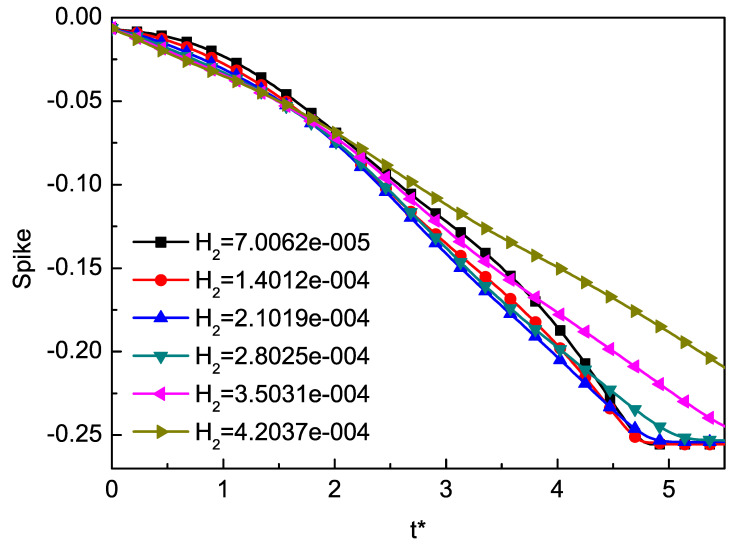
The time evolutions of the spike at different values of Knudsen number $H_{2}$. The Knudsen number tends to inhibit the RT instability.

**Figure 4:**
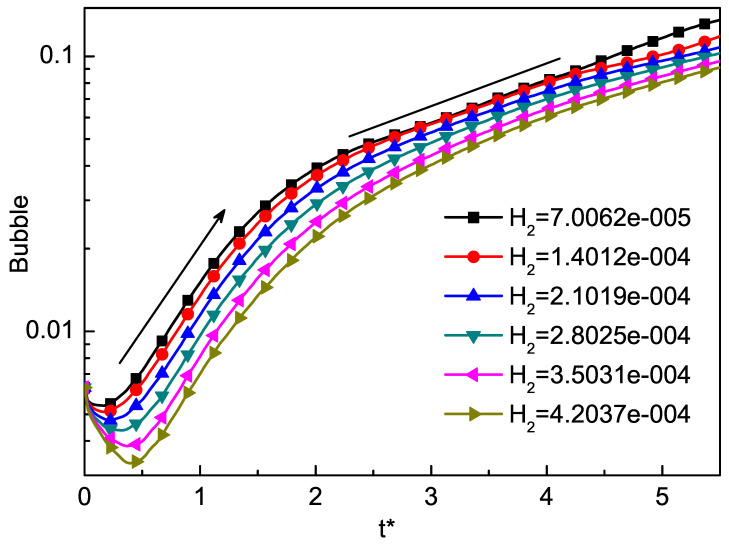
The growth of the bubble by the DBM. The good linear fit is consistent with the growth of the bubble at the beginning.

**Figure 5:**
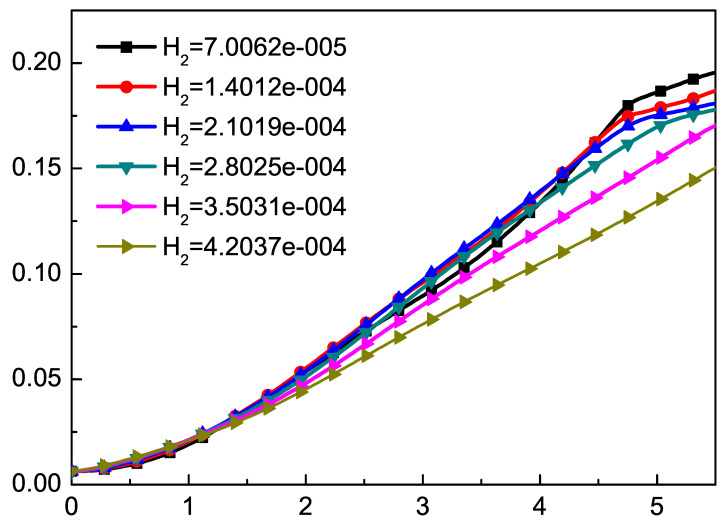
The growth of the amplitude with different values of the Knudsen number. The effect of $H_{2}$ in the prophase is not significant, while later the effect gradually emerged.

In general, the thickness of the mixed layer is an important parameter to measure the evolution of RT instability. We track the positions of bubble and spike as an indicator of the mixed layer, and define half the distance between the bubble and the spike as the amplitude. In order to study the effect of $H_{2}$ on RT instability, we use the local TNE quantity $\Delta_{(3,1)-y}^{*}$ to track the position of spike and bubble [74]. In order to study the effect of Knudsen number on the RT instability of compressible fluid, we can adjust the dimensionless Knudsen number $H_{2}$ through τ by Equation (34). We choose six different $H_{2}$ values $7.0062\times 10^{-5}$, $1.4012\times 10^{-4}$, $2.1019\times 10^{-4}$, $2.8025\times 10^{-4}$, $3.5031\times 10^{-4}$, $4.2037\times 10^{-4}$, corresponding to six different τ as $1\times 10^{-5}$, $2\times 10^{-5}$, $3\times 10^{-5}$, $4\times 10^{-5}$, $5\times 10^{-5}$, $6\times 10^{-5}$. The results are shown in Figure 3 and Figure 4. Here, $t^{*}=t/\sqrt{2\pi /(gk)}$. Figure 3 displays the time evolutions of the spike with the dimensionless Knudsen number $H_{2}$. From the evolution of the spike, we can find that the Knudsen number tends to inhibit the RT instability. Figure 4 displays the time evolutions of the bubble with the dimensionless Knudsen number $H_{2}$. It is interesting to observe that, when $t^{*}<0.5$, with the increase of $t^{*}$, the larger $H_{2}$, the faster it goes down. Meanwhile, from $t^{*}=0.5$ to $t^{*}=1.5$ and $t^{*}=2.5$ to $t^{*}=3.5$, the bubble grows exponentially, and from $t^{*}=0.5$ to $t^{*}=1.5$, the growth rate is faster than from $t^{*}=2.5$ to $t^{*}=3.5$. This phenomenon can be understood as first the system in the adjustment phase, then the stored compressive energy of the system is released into kinetic energy. As the Kundsen number gets larger, the collision time of particles in the system gets longer, and the conversion between energies also gets longer. Figure 5 shows the growth of the amplitude with different values of the Knudsen number. The effect of $H_{2}$ in the prophase is not significant, while later the effect gradually emerged. It can be found that the effects of Knudsen number stabilize the RT instability as a whole.

**Figure 6:**
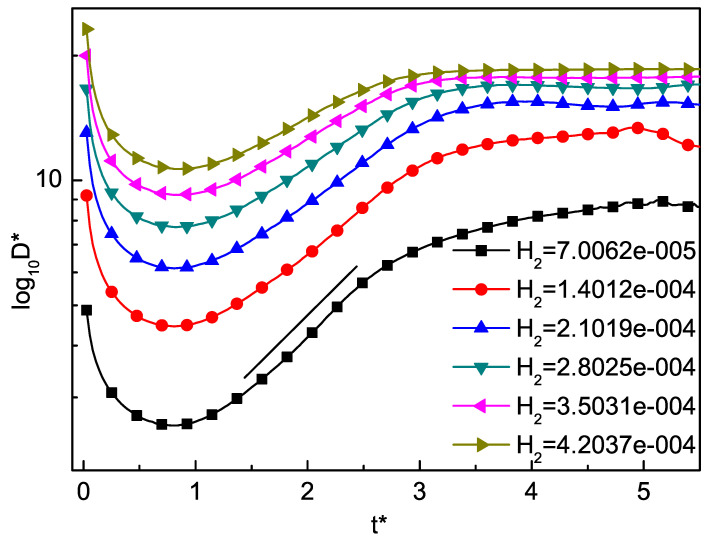
The time evolution of the total average TNE strength with different values of Knudsen number $H_{2}$. The total average TNE strength first decreases in the first stage and then increases in the later stage for each $H_{2}$. The higher the Knudsen number, the stronger the total average TNE effects.

The time evolution of the total average TNE strength of the RT instability with different values of Knudsen number $H_{2}$ are shown in Figure 6. It is interesting to find that the total average TNE strength first decreases in the first stage and then increases in the later stage for each $H_{2}$. Furthermore, the higher the Knudsen number, the stronger the total average TNE effects. Note that, as $H_{2}$ is increased, the linear stage of growth is prolonged in time, then the total average TNE strength tends to a constant.

**Figure 7:**
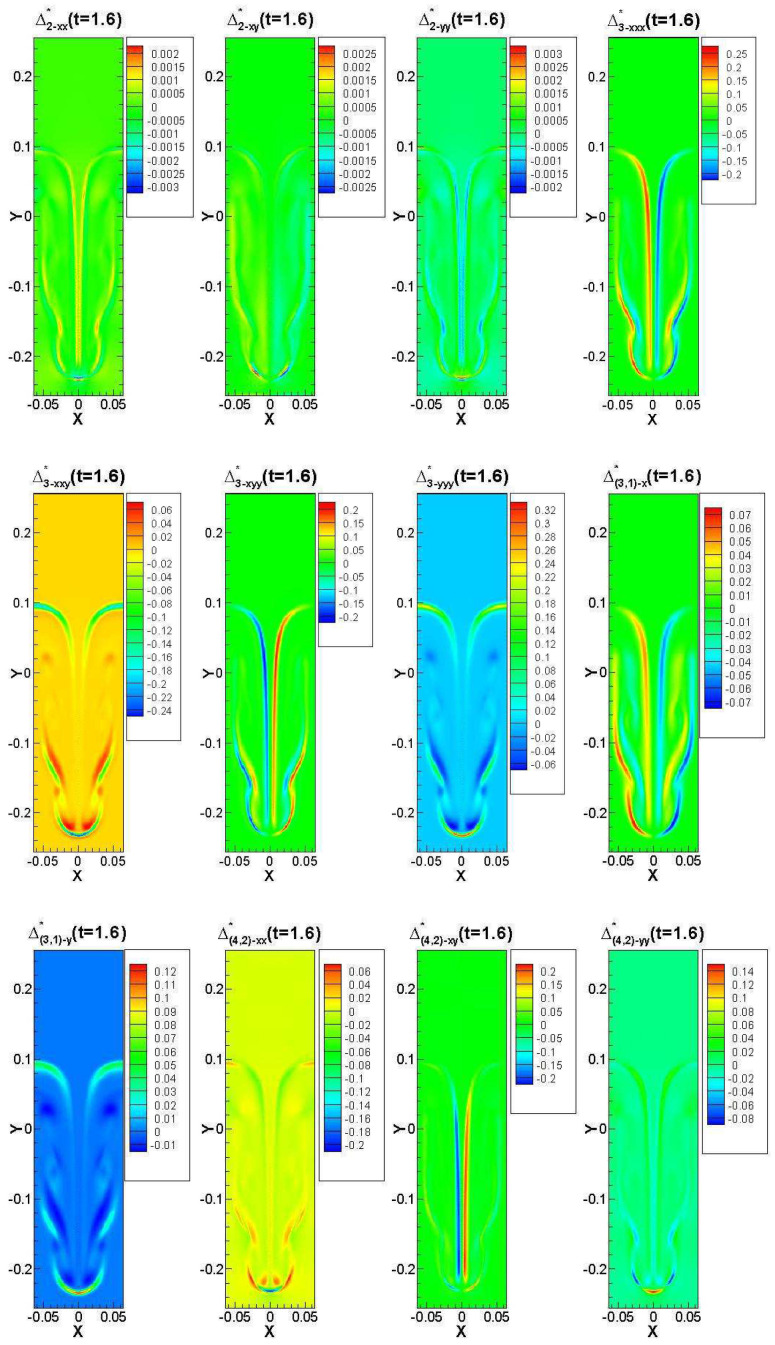
Contours of various components of TNE effects at t=1.6 under the condition of $H_{2}=1.4012\times 10^{-4}$. The TNE effects are mainly concentrated on the two sides of the interface, far away from the interface, and the TNE effect is basically zero. Some of the TNE effects are relatively smaller, some are larger, and some showed the opposite effect.

In Figure 7, we illustrate the profiles of each TNE quantity. We find that the description of non-equilibrium characteristics is not identical. Each TNE component is embodied in a certain degree of freedom, and different degrees of freedom have different physical meanings. $\Delta_{2-xx}^{*}$ is the degree to which the internal energy of *x* degrees of freedom deviates from the equilibrium state, $\Delta_{2-xy}^{*}$ represents the non-equilibrium characterization associated with the shear effect, $\Delta_{3-xxy}^{*}$ represents the flux of the internal energy in the *x* direction in the *y* degrees of freedom, $\Delta_{3-yyy}^{*}$ represents the flux of internal energy in the *y*-direction at *y* degrees of freedom, $\Delta_{(3,1)-x}^{*}$ represents the internal energy flux in the *x* direction, $\Delta_{(3,1)-y}^{*}$ represents unorganized energy flow in the *y* direction, and $\Delta_{\left(4,2\right)}^{*}$ represents the flux of internal energy flux. Far from the interface, the value of non-equilibrium strength is basically zero, and the non-equilibrium effect is mainly concentrated on both sides of the interface, which is closely related to the gradient of macroscopic quantities. Near the interface, the TNE effect is obvious and the intensity is large, indicating that the system at the interface deviates far from the thermodynamic equilibrium state. DBM provides an effective tool for the study of TNE non-equilibrium phenomena beyond the description of traditional hydrodynamic models, which just provides only the viscosity and the heat conduction effect.

## 4. Conclusions

In this paper, based on our previous work [74], we continue to use the discrete Boltzmann method (DBM) to study the two-dimensional (2D) Rayleigh–Taylor (RT) instability in the compressible fluids. In the framework of our model, there are two dimensionless parameters, one represents the compressibility $H_{1}$, and the other represents the Knudsen number $H_{2}$. We focus on the latter. The hydrodynamic non-equilibrium (HNE) effects and the thermodynamic non-equilibrium (TNE) effects of the compressible RT instability are investigated by changing the non-dimensional parameter Knudsen number $H_{2}$ when the compressibility doesn’t change. 2D HNE and TNE effects are demonstrated in detail. We also investigate the “spike” and “bubble” of the 2D RT instability traced by the local non-equilibrium quantity $\Delta_{(3,1)-y}^{*}$. The effects of the Knudsen number $H_{2}$ on the developments of RT instability are carefully analyzed for six cases with the same initial perturbations. From the simulation results, we find that the Knudsen number $H_{2}$ plays an important role in the RT instability. The mixing-zone grows inversely with increasing the Knudsen number $H_{2}$. At the same time, the larger $H_{2}$, the more obvious the Kelvin–Helmholtz instability for the later development, and the faster the shape of the mushroom appears. Furthermore, different TNE quantities display a wealth of non-equilibrium information. Far from the disturbance interface, the value of non-equilibrium strength is basically zero, and the non-equilibrium effect is mainly concentrated on both sides of the interface, which is closely related to the gradient of macroscopic quantities. Near the interface, the various TNE effects are more obvious and the corresponding intensity are relatively large, indicating that the interface of the system deviates far from the thermodynamic equilibrium state. The observations on the HNE and TNE effects influenced by the effects of Kundsen number are helpful for a better understanding the process of RT instability on compressible fluids.
